# Protection against Aβ-induced neuronal damage by KU-32: PDHK1 inhibition as important target

**DOI:** 10.3389/fnagi.2023.1282855

**Published:** 2023-11-14

**Authors:** Ranu Pal, Dongwei Hui, Heather Menchen, Huiping Zhao, Olivier Mozziconacci, Heather Wilkins, Brian S. J. Blagg, Christian Schöneich, Russell H. Swerdlow, Mary L. Michaelis, Elias K. Michaelis

**Affiliations:** ^1^Higuchi Biosciences Center, University of Kansas, Lawrence, KS, United States; ^2^Department of Pharmaceutical Chemistry, University of Kansas, Lawrence, KS, United States; ^3^Department of Medicinal Chemistry, University of Kansas, Lawrence, KS, United States; ^4^University of Kansas Alzheimer’s Disease Research Center, University of Kansas Medical Center, Kansas City, KS, United States; ^5^Department of Chemistry and Biochemistry, University of Notre Dame, South Bend, IN, United States

**Keywords:** Novobiocin analog, superoxide, mitochondria, complex I, pyruvate dehydrogenase kinase

## Abstract

A feature of most neurodegenerative diseases is the presence of “mis-folded proteins” that form aggregates, suggesting suboptimal activity of neuronal molecular chaperones. Heat shock protein 90 (Hsp90) is the master regulator of cell responses to “proteotoxic” stresses. Some Hsp90 modulators activate cascades leading to upregulation of additional chaperones. Novobiocin is a modulator at the C-terminal ATP-binding site of Hsp90. Of several novobiocin analogs synthesized and tested for protection against amyloid beta (Aβ)-induced neuronal death, “KU-32” was the most potent in protecting primary neurons, but did not increase expression of other chaperones believed to help clear misfolded proteins. However, KU-32 reversed Aβ-induced superoxide formation, activated Complex I of the electron transfer chain in mitochondria, and blocked the Aβ-induced inhibition of Complex I in neuroblastoma cells. A mechanism for these effects of KU-32 on mitochondrial metabolism appeared to be the inhibition of pyruvate dehydrogenase kinase (PDHK), both in isolated brain mitochondria and in SH-SY5Y cells. PDHK inhibition by the classic enzyme inhibitor, dichloroacetate, led to neuroprotection from Aβ_25-35_-induced cell injury similarly to KU-32. Inhibition of PDHK in neurons would lead to activation of the PDH complex, increased acetyl-CoA generation, stimulation of the tricarboxylic acid cycle and Complex I in the electron transfer chain, and enhanced oxidative phosphorylation. A focus of future studies may be on the potential value of PDHK as a target in AD therapy.

## 1. Introduction

A characteristic of Alzheimer’s disease (AD) and other age-related, neurodegenerative diseases, is the accumulation of oligomers or aggregates of misfolded proteins in or around neuronal cells. In AD, there is extracellular accumulation of and plaque formation by β amyloid peptide (Aβ), and intracellular fibril formations containing misfolded, hyper-phosphorylated TAU (p-TAU) proteins (Muchowski, 2002; Muchowski and Wacker, 2005; Gidalevitz et al., 2006). The cell stress caused by p-TAU or Aβ aggregates leads to aggregation of well over 100 other proteins in neurons (Pace et al., 2018a,b). The presence of such aggregates of misfolded proteins in aging-related neurodegenerative diseases and animal models of such diseases, suggests that the cellular protein quality control machinery, including the endosomal-lysosomal network, may be compromised in the aging brain, making brain cells more vulnerable to “proteotoxicity” (Arslan et al., 2006; Gidalevitz et al., 2010; Nixon, 2017).

Efforts to target faulty protein folding in neurodegenerative diseases have focused frequently on the protein chaperone, heat shock protein 90 (HSP90), the most abundant HSP and a central regulator of responses to a wide array of cell stresses (Peterson and Blagg, 2009; Taipale et al., 2010; Bohush et al., 2019). Inhibitors of the ATP binding site in the *N*-terminal region of HSP90, generally, lead to the release of heat shock factor 1 (HSF1) from HSP90, the subsequent activation and translocation of HSF1 into the nucleus, and the initiation of the “heat shock response” (HSR), or “proteotoxic stress response” (Neckers, 2002; Dai and Sampson, 2016; Dayalan Naidu and Dinkova-Kostova, 2017). The HSR is characterized by induction of chaperones, such as HSP70 and HSP40, that aid in re-folding or clearance of aggregated proteins (Dou et al., 2003; Waza et al., 2005; Dickey et al., 2006; Chen et al., 2014; Dai and Sampson, 2016; Dayalan Naidu and Dinkova-Kostova, 2017).

There is ample evidence that in the nervous system, HSP90 and specific client proteins are involved in the regulation of the levels of pTAU (Shimura et al., 2004; Dickey et al., 2007; Luo et al., 2007) and that inhibition of HSP90 causes decreases in pTAU (Dickey et al., 2007; Luo et al., 2007). It is noteworthy that HSP90 inhibitors decrease pTAU levels without causing the release of HSF1 and the initiation of the HSR (Dickey et al., 2007). Alternative processes activated in neurons following HSP90 inhibition include decreases in pTAU levels due to either suppression of the activity of TAU kinases (Luo et al., 2007) or increases in degradation of pTAU by co-chaperones or client proteins of HSP90 (Dickey et al., 2007).

Detailed analysis of the proteomic response of cells in select brain regions of Tau transgenic mice or in neuronal cells in culture overexpressing TAU, showed that the stress of TAU accumulation leads to a rearrangement of proteomic connectivity among HSP90, HSP90 co-chaperones, client proteins, and other interacting proteins (Inda et al., 2020). The proteome of interacting proteins included proteins important to cell metabolism, translation, transcription, synaptic signaling, and other neuronal functions (Inda et al., 2020). These changes represent abnormal realignments of protein interactions in the HSP90-related proteome that revert to normal connectivity patterns following inhibition of HSP90.

The HSP90 inhibitors used in the studies described above are those that target the *N*-terminal ATPase domain of HSP90 (Neckers et al., 1999; Huck et al., 2019). A series of HSP90 inhibitors have also been designed that interact with the *C*-terminal domain and are based on the structure of novobiocin, an inhibitor of proteins with an ATP-binding domain, the GHKL domain, common to gyrases, histidine kinases, MutL, and HSP90 (Peterson and Blagg, 2009; Matts et al., 2011; Simon et al., 2017). A library of novobiocin analogs was synthesized and used to screen for protective *vs* cytotoxic effects in primary neurons and neuronal cell lines (Yu et al., 2005) and a non-toxic analog of novobiocin (designated “A4”) was identified as being protective of neurons against Aβ – induced stress and toxicity *in vitro* and showed potential to cross the blood brain barrier (Ansar et al., 2007; Lu et al., 2009). Several studies point to the synergistic effects of Aβ and TAU on neurodegeneration (Busche and Hyman, 2020), thus neuroprotection from Aβ-induced stress probably involved suppression of the TAU-HSP90 proteome changes described above. Subsequently, two novobiocin analogs designated “KU-32” and KU-596, were shown to protect dorsal root ganglion neurons (DRG), both *in vitro* and *in vivo*, from the damage induced by experimental diabetic peripheral neuropathy (Ma et al., 2014, 2015; Anyika et al., 2016).

Both KU-32 and KU-596 produce their effects on DRG diabetic neuropathy through activation of the HSR in an HSP70-dependent manner (Ma et al., 2014, 2015; Anyika et al., 2016). But, as described above, neuroprotection against pTAU-related pathology in central nervous system (CNS) neurons is shown to be independent of HSF1-initiated HSR (Dickey et al., 2007). Therefore, it would be important to know whether KU-32 and KU-596 offer protection to brain neurons from the stress and damage induced by Aβ and pTAU, and to define the mechanisms for such neuroprotection.

In the present studies, we tested both KU-32 and KU-596 for neuroprotection from the cell damaging effects of Aβ-induced toxicity in primary cultures of rat cortical neurons, and found KU-32 to be completely neuroprotective at low nanomolar concentrations, and KU-596 partially protective at micromolar concentrations. Unlike the induction of HSP70 observed following treatment of cortical neurons with the *N*-terminal inhibitor geldanamycin (GA), KU-32 did not lead to the induction of HSP70. Metabolic stress resulting from mitochondrial dysfunction in brain neurons has been proposed as an alternative mechanism for neuronal stress following exposure to Aβ or in response to aggregated TAU, and we identified the mitochondrial enzyme pyruvate dehydrogenase kinase (PDHK) to be a target of KU-32 and linked to neuroprotection by KU-32.

## 2. Methods

### 2.1. Synthesis of KU-32 and KU-596 and the preparation of the Aβ-peptides

The synthesis and determination of structural purity of KU-32 and KU-596 were as described previously (Burlison and Blagg, 2006; Donnelly et al., 2008; Kusuma et al., 2012; Anyika et al., 2016). KU-32 or KU-596, dissolved in either Captisol^®^ (Ligand Pharmaceuticals, LaJolla, CA) or DMSO, were used to treat cortical neurons or SH-SY5Y neuroblastoma cells (final concentrations of Captisol and DMSO in cell culture were 0.05 and 0.02%, respectively). The Aβ_1-42_ and Aβ_25-35_ peptides (Anaspec, Freemont, CA) were suspended in sterile 50 mM Tris–HCl, pH 7.4, at 1 mM concentration and stored in small aliquots at −20°C. Application of Aβ_1-42_ or Aβ_25-35_ peptides to neuronal cultures followed incubation of the peptide solutions at 37°C for 24 h to promote oligomerization.

### 2.2. Experimental animals

Procedures related to animals (C57Bl6 mice, Sprague–Dawley female rats, and mixed gender rat pups) followed those of the Institutional Animal Care and Use Committee (IACUC) of the University of Kansas (IACUC # 40–09, 39–02).

### 2.3. Preparation of primary rat cortical neurons and exposure to Aβ, KU-32, or KU-596

The brains of embryonic day-18 rats of unknown gender were the source of cortical neurons (Michaelis et al., 1998, 2005). The cells were re-suspended in Dulbecco’s modified Eagle’s medium/F12 (DMEM; Sigma-Aldrich, St. Louis, MO.) plus 10% fetal bovine serum (FBS; Atlanta Biologicals, Lawrenceville, GA) and plated at a density of 2.5 × 10^5^ cells in 35 mm glass-bottom dishes (Mat-Tek Co., Ashland, MA) coated with 10 μg/mL poly-D-lysine and 5 μg/mL laminin. After 24 h, the serum-containing medium was removed, the neurons maintained in Neurobasal medium with 2% B-27 supplements (Invitrogen, Carlsbad, CA), and grown at 37°C (5% CO_2_) for 7 days. Two h before addition of either Aβ_1-42_ or Aβ_25-35_ (10 μM, final concentration), the cultures were treated with either vehicle or the indicated concentrations of KU-32 or KU-596. After the addition of the Aβ peptides, the primary neurons were incubated for 48 h. The effects of Aβ on the viability of primary neurons in the presence or absence of KU-32 or KU-596 were determined by monitoring cell survival using the Live-Dead assay (Michaelis et al., 1998, 2005). Following labeling of neurons with 20 μM propidium iodide and 150 nM calcein acetoxy-methylester (Invitrogen, Carlsbad, CA), the number of viable and dead cells were counted in captured digital images from six fields per dish. All experiments were conducted using duplicate dishes from three neuronal preparations for each treatment (~1,000 cells analyzed per treatment condition) and the data expressed as the fraction of viable cells calculated from the total number of neurons counted under each treatment condition.

### 2.4. HSP70 levels following treatment with KU-32 or GA

To determine the levels of HSP70 in primary neurons following 24 h treatment with varying concentrations of KU-32 or GA (EMD Millipore, Billerica, MA), the cells were lysed, protein concentration measured (BCA assay, ThermoFisher, Waltham, MA), equal amounts of protein per lane separated by SDS-PAGE, transferred to PVDF membranes, and probed with an antibody to inducible HSP70 (Enzo, Farmingdale, NY). Actin labeling by anti-actin antibodies (Santa Cruz Biotechnology, CA) served as a loading control.

### 2.5. Superoxide (O_2_^•-^) levels in SH-SY5Y cells

The intracellular formation of O_2_^•-^ as an index of the generation of ROS following treatment of undifferentiated neuronal SH-SY5Y cells (American Tissue Culture Collection) with Aβ_25-35_ or KU-32 was measured by monitoring the formation of 2-hydroxyethidium (2-OH-E^+^) as a product of the reaction of hydroxyethidine (HE) with O_2_^•-^ (Zhao et al., 2005). The SH-SY5Y cells were grown to >80% confluence in a medium consisting of 1:1 advanced DMEM F12 supplemented with 10% FBS. The cells were subsequently treated with either KU-32 (200 nM), Aβ_25-35_ (10 μM), the combination of the two, or only vehicle, incubated for 22 h at 37° C, rinsed with PBS, suspended in PBS, incubated with 20 μM HE for 30 min, harvested by centrifugation (1,000 *g* × 2 min), and frozen at −80°C. After thawing, the cells were lysed and protein levels were measured prior to extraction of HE and 2-OH-E^+^ in butanol as described (Zhao et al., 2005). HE and 2-OH-E^+^ were separated by HPLC (C18 column; Vydac 218TP54, 5 μm, 4.6 × 250 mm) on a Shimadzu system with an RF-20A fluorescence detector, and detected by fluorescence (ex 510 nm; em 595 nm; Zielonka et al., 2008).

### 2.6. Mitochondrial complex I activity in neuroblastoma SH-SY5Y cells

Undifferentiated SH-SY5Y cells (>80% confluent) were treated with 10 μM Aβ_25-35_, 200 nM KU-32, the combination of KU-32 and Aβ, or vehicle, and incubated for 48 h. Mitochondria were isolated from these cells as described (Frezza et al., 2007). Enrichment of the final pellet with mitochondria confirmed by immunoblots for VDAC, NDUFB8, and TRAP1 (Supplementary Figure). Antibodies used: anti-VDAC (ThermoFisher, 1:250), anti-NDUFB8 (Novus Biologicals, Littleton, CO, 1:500), anti-TRAP1 (abcam, ab182775). Complex I (NADH:ubiquinone oxidoreductase) activity in mitochondria was measured as described (Janssen et al., 2007). The mitochondria were ruptured by freeze–thaw cycles, and 10 μg mitochondrial protein incubated (3 min, 37°C) in 25 mM potassium phosphate buffer (pH 7.4) containing 3.5 mg/mL BSA, 60 μM 2,6-dichlorophenol-indophenol, 70 μM decylubiquinone, and 1 μM antimycin A in 96-well plates. NADH added (0.2 mM final concentration) and the absorbance at 590 nm measured at 30-s intervals for 4 min. After 4 min, rotenone (1 μM final concentration) added to inhibit Complex I and the absorbance measured at 30-s intervals for 4 min. A transformation of the integrated Michaelis equation V_app_•t = ([S_0_] – [S]) + (K_M_ • ln([S_0_]/[S]); Wharton and Szawelski, 1982) was used to estimate the V_max_ and K_M_ from the rate kinetics of Complex I.

### 2.7. PDHK activity in brain mitochondria and SH-SY5Y cell homogenates

The PDHK activity in mouse brain mitochondria or SH-SY5Y cell homogenates was the net ATP-dependent suppression of PDH complex (PDHC) activity (Sheu et al., 1984; Butterworth, 1989). The brains from mice were removed following decapitation, specific regions were dissected (Bao et al., 2009), and each region homogenized in buffered 0.32 M sucrose/MgSO_4_ medium containing protease inhibitors (Michaelis et al., 1983). The homogenates were aliquoted to tubes (25 μL/tube), rapidly frozen in liquid N_2_ and stored (−80°C). Two to four tubes of homogenates from the cerebellum and cortex were thawed at 23°C, diluted (1:10) with a KCl buffer (Kerbey et al., 1977), and the mitochondria isolated by differential centrifugation (Fatania et al., 1986). To activate the PDHC, the mitochondrial pellets were re-suspended in Tris-MOPS buffer containing (in mM): 20 Tris-MOPS, 1 dithiothreitol (DTT), 5 MgCl_2_, 0.1 CaCl_2_, plus 0.1% Triton X-100 (Sheu et al., 1984). To triplicate 50 μL mitochondrial samples (20–30 μg protein/50 μL) were added either 200 nM KU-32 in dimethyl sulfoxide (DMSO), 10 mM dichloroacetic acid (DCA) in DMSO, or buffer with DMSO (0.2% DMSO final concentration in all samples), and the PDHC was activated by incubating the samples at 30°C for 15 min. At the end of incubation, the samples were quickly frozen, and stored overnight at −80°C. To activate the PDHK, the samples were thawed at 23°C, transferred into wells of a 96 well plate and 50 μL of 80 mM Tris-MOPS activation buffer with or without 4 mM ATP was added (Butterworth, 1989). Following incubation at 30°C for 2 min in a Biotek Synergy HT plate reader, the reaction was quenched with 50 μL of PDHC assay mixture containing (in mM): 50 potassium phosphate buffer, 1 MgCl_2_, 2.5 NAD, 0.2 thiamine pyrophosphate (TPP), 0.1 coenzyme A (CoA), 0.3 DTT, 0.6 2(*p*-iodophenyl)-3-*p*-nitrophenyl-tetrazolium chloride (INT), plus 0.0065 phenazine methosulfate. Enzyme activity was initiated by the addition of 5 mM pyruvate and the formation of NADH (ΔA at 490 nm) measured every 30 s for 10 min (Butterworth, 1989).

### 2.8. PDHK phosphotransfer activity measurement

Direct measurements of PDHK phosphorylation of PDHC were conducted using the sandwich enzyme-linked immunosorbent assay (ELISA) that detects the phosphorylation of S232 in PDH E1α1 subunit of PDHC (Abcam, Cambridge, United Kingdom). Mitochondria from mouse brain cerebellum were isolated and treated for activation of PDHK in the presence of either KU-32 or vehicle as described above. Following induction of PDHC phosphorylation in the presence of Mg-ATP, the measurement of the level of phospho-S232 in PDH E1α was according to the manufacturer’s protocol.

### 2.9. Intracellular and mitochondrial distribution of Aβ peptides

SH-SY5Y cells grown on glass “cut-out” dishes, were incubated for 18 h with AlexaFluor-derivatized 1 μM Aβ_1-40_ HiLyte Fluor 488 (Anaspec, Freemont, CA). The medium was removed, 1 μM MitoTracker Orange (ThermoFisher) in culture medium added, the cells incubated for 30 min (37°C), washed, and then fixed with paraformaldehyde. Following washing, the cells were incubated for 24 h at 23°C with 4,6 diamidino-2-phenylindole (DAPI) and the samples visualized in a Leica confocal microscope.

### 2.10. Assessment of SH-SY5Y cell injury

The cell-damaging effects of Aβ on SH-SY5Y cells, in the presence or absence of KU-32 or DCA, were measured as the release of lactate dehydrogenase (LDH) employing the coupled reaction of LDH/diaphorase exactly as described in the protocol (*mcb.berkeley.edu/labs/krantz/protocols/LDH%20Assay%20Protocol.doc*).

### 2.11. Statistical analyses

Data from all assays represent means and standard errors of the mean (SEM). Statistical methods used to estimate significant differences (*p* ≤ 0.05) were one-way ANOVA with Bonferroni *post-hoc* analysis or paired or unpaired *t*-test for two-group comparisons.

## 3. Results

### 3.1. KU-32 and KU-596 protection from Aβ exposure of cortical neurons in culture

**Figure 1:**
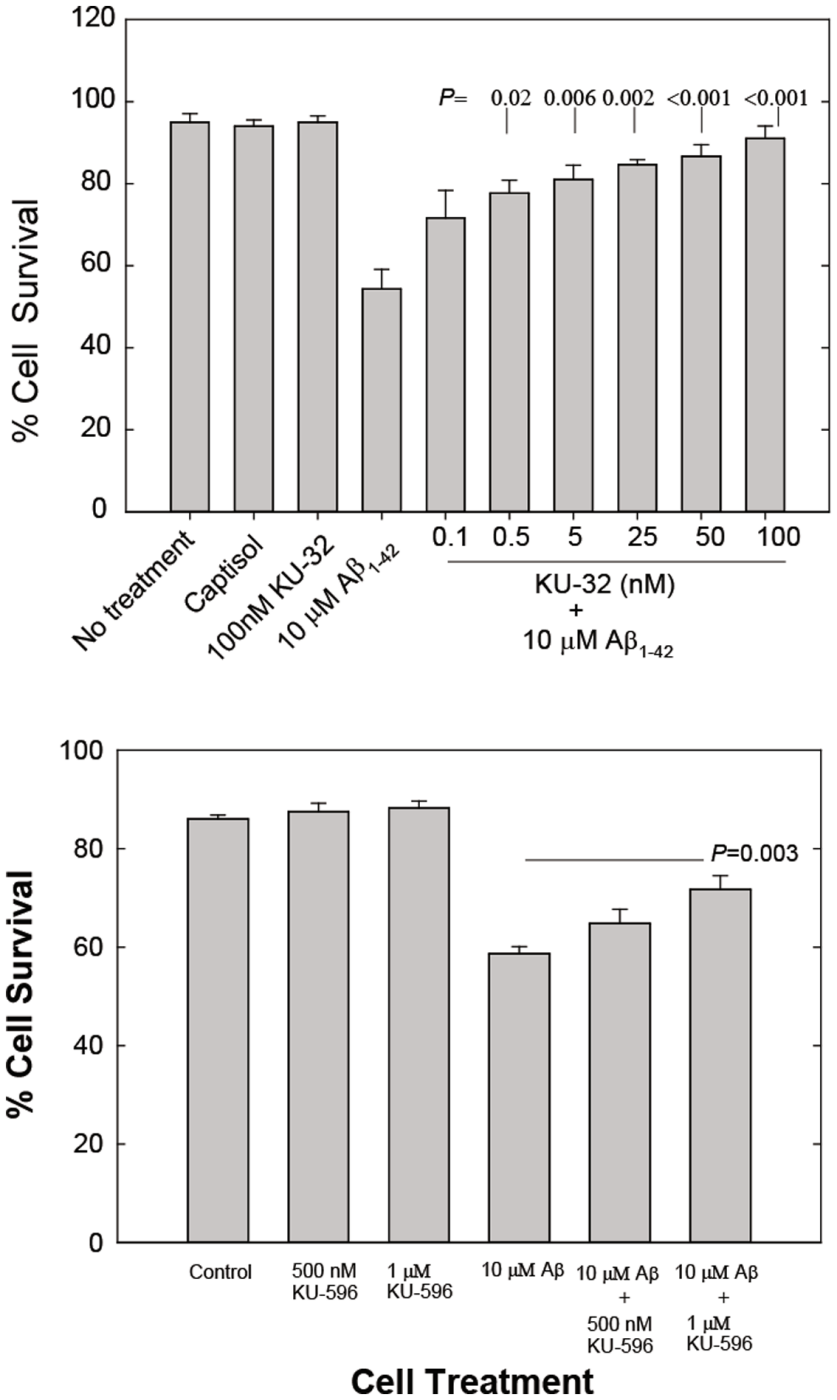
**(A)** Concentration-dependent effects of KU-32 on Aβ_1-42_-induced cell death in primary cortical neurons. Cultures were either treated with vehicle (Captisol), 100 nM KU-32, 10 μM Aβ_1-42_, or pretreated for 2 h with varying concentrations of KU-32 and then with 10 μM Aβ. Neuronal survival at 48 h post-treatment measured as described under Methods. The data represent the mean percentage (± SEM) of surviving neurons in six fields/dish from three culture preparations (~1,000 cells/condition). Statistically significant differences between treatment with Aβ only and cultures pretreated with KU-32 followed by Aβ are shown as the respective *p* values. **(B)** Concentration-dependent effects of KU-596 on Aβ-induced cell death in cortical neurons. Assay conditions were identical to those in **(A)** and statistically significant differences between Aβ_1-42_-treated and Aβ plus KU-596 treated cell cultures shown.

Exposure of primary embryonic rat cortical neurons to 10 μM Aβ_1-42_ caused stress that typically led to the death of approximately 50% of the neurons within 48 h (Figure 1A). The addition of 0.1 nM to 100 nM KU-32 2 hours prior to the addition of the Aβ peptide protected cortical neurons against cell death in a concentration-dependent manner (Figure 1A). Pre-treatment of rat primary cortical neurons with KU-32 produced nearly identical neuroprotection from the toxicity of Aβ_25-35_ (data not shown). The EC_50_ for protection of neurons from Aβ_1-42_ by KU-32 was estimated to be approximately 1 nM. Treatment of neurons with 100 nM KU-32 offered nearly complete protection from the stress induced by Aβ_1-42_. The addition of 100 nM KU-32 alone produced no detectable toxicity in the neuronal cultures (Figure 1A).

The novobiocin analog KU-596, designed to optimize the fit of the molecule to the HSP90 *C*-terminal site to which novobiocin binds, protects DRG neurons and cells in a neuronal cell line from the stress and toxicity produced by elevated glucose levels (glucotoxicity) with an estimated EC_50_ of 13 nM (Kusuma et al., 2012). KU-32 is not as effective as KU596 in protecting DRG neurons (EC_50_ = 240 nM; Kusuma et al., 2012). However, with respect to protection of primary rat cortical neurons, low nanomolar concentrations of KU-596 offered no protection against Aβ_1-42_-induced stress and toxicity (data not shown) and concentrations of 500 nM and 1 μM offered only partial neuroprotection (25 and 48% more surviving neurons, respectively; Figure 1B). KU-596 when introduced alone did not affect neuronal viability (Figure 1B). All subsequent studies described in this report focused on the characterization of possible mechanisms of neuroprotection from Aβ-induced toxicity by the more potent of the novobiocin analogs tested, KU-32.

### 3.2. Lack of effect of KU-32 On HSP70 levels in primary cortical neurons

**Figure 2:**
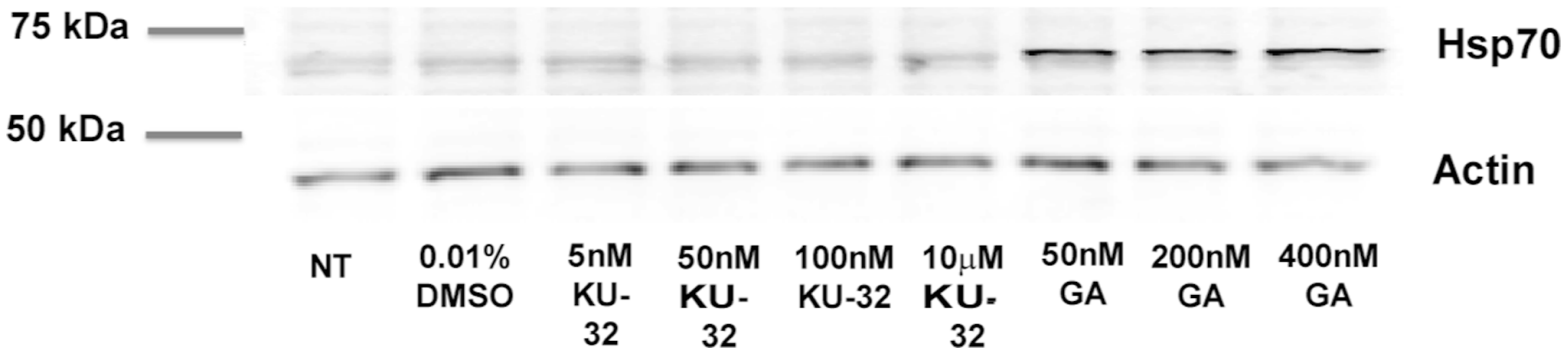
Effects of KU-32 and GA on Hsp70 levels in primary neurons. Neurons at 7 days *in vitro* treated with vehicle (DMSO, 0.01%), KU-32, or GA at the concentrations indicated, and the cell lysates subjected to SDS-PAGE and immunoblotting with anti-Hsp70 and anti-actin antibodies (NT: no treatment). Optical densities of Hsp70 bands were: NT- 38; DMSO –54; 5 nM KU-32–56; 50 nM KU32–50; 100 nM KU-32–53; 10 μM KU-32–60; 50 nM GA–222; 200 nMGA–186; 400 nM GA–236. The experiment repeated twice with similar results.

We first determined the efficiency of KU-32 in altering HSP70 levels in cortical neurons as a measure of possible induction of the HSR. We probed for evidence of HSP70 induction by using primary neuronal cultures treated with varying concentrations of either KU-32 or geldanamycin (GA) as shown in Figure 2. Exposure of neurons to KU-32 at concentrations between 5 nM and 10 μM had no apparent effect on the levels of Hsp70 (optical densities of antibody-labeled bands of KU-32-treated cells were in the same range as vehicle controls), whereas all concentrations of GA tested produced approximately fourfold increases in HSP70 in neurons (Figure 2). Thus, in cortical primary neurons, KU-32 offered neuroprotection against Aβ toxicity in the absence of an effect on HSP70-mediated HSR.

### 3.3. KU-32, Aβ, and O_2_^•-^ levels in SH-SY5Y cells

To explore alternative molecular pathways for the protection by KU-32 of neurons under Aβ-induced stress, we used the model of the neuroblastoma cell line, SH-SY5Y, and the stress induced in these cells by exposure to Aβ. The SH-SY5Y cells are susceptible to the toxicity of Aβ_1-42_ and Aβ_25-35_, as both Aβ peptides at micromolar concentrations induce cell death (Lu et al., 2009; Xu et al., 2009). We first examined whether exposure to Aβ induces an increase in the levels of O_2_^•-^ in SH-SY5Y cells, as a measure of oxidative stress, and that the neuroprotection by KU-32 may be the result of decreased Aβ-induced O_2_^•-^ formation. In these and subsequent studies examining the activity of KU-32 in SH-SY5Y cells or on enzymes we employed a KU-32 concentration of 200 nM, i.e., a concentration that would be expected to produce maximal neuroprotection.

**Figure 3:**
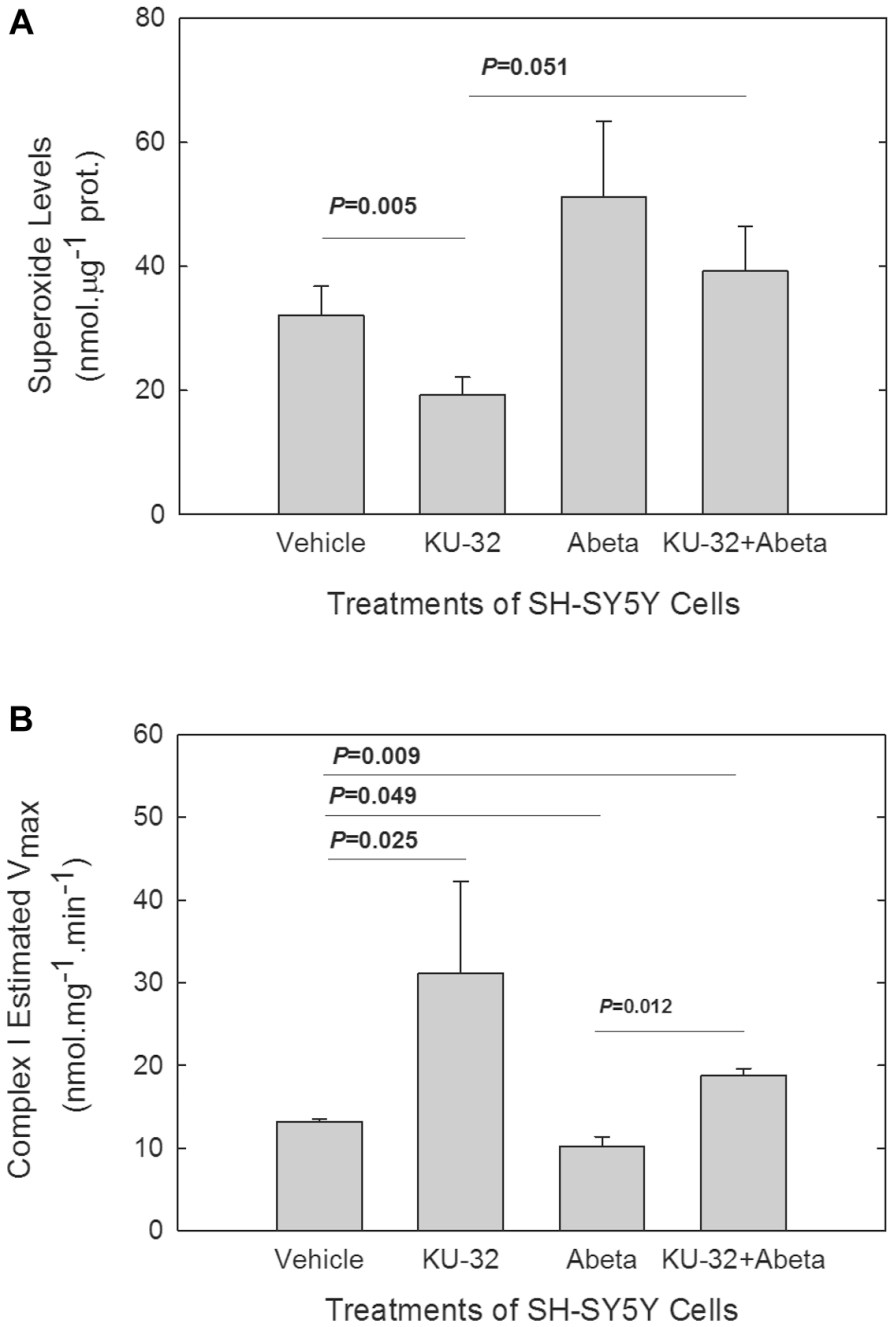
Effects of Aβ and KU-32 on O_2_^•-^ levels in SH-SY5Y cells **(A)** and on Complex I of the ETC activity **(B)** in mitochondria isolated from treated cells. **(A)** Superoxide levels (mean ± SEM) in SH-SY5Y cells treated for 22 h with vehicle (DMSO), 200 nM KU-32, 10 μM Aβ_25-35_, or KU-32 plus Aβ (*n* = 5 cultures/treatment condition, run in parallel). Values of *p* significant differences (*t*-test) shown. **(B)** Mean V_max_ values (± SEM) for Complex I of mitochondria isolated from six cultures of SH-SY5Y per treatment condition. The SH-SY5Y cells were treated for 48 h with either vehicle (DMSO), 200 nM KU-32, 10 μM Aβ_25-35_, or KU-32 plus Aβ as described above. *p* values of significant differences in V_max_ shown (*t*-test).

The O_2_^•-^ concentration in cells was measured following *in vitro* treatment with Aβ_25-35_ in cells that were pre-treated with either KU-32 or vehicle. Treatment of cells with 200 nM KU-32 alone produced a significant decrease in the levels of O_2_^•-^ as compared with those in vehicle-treated cells (Figure 3A). Treatment with 10 μM Aβ_25-35_ brought about a rise, yet not significant, in cellular O_2_^•-^. Treatment of SH-SY5Y cells with KU-32 prior to the introduction of Aβ reduced the Aβ-induced rise in O_2_^•-^ levels, but did not bring the O_2_^•-^ levels to those of vehicle-treated cells (Figure 3A). Therefore, the overall effect of Aβ was an increase in O_2_^•-^ whereas that of KU-32 was a reduction in O_2_^•-^ levels in neuronal cells.

### 3.4. KU-32-induced increase and Aβ decrease of the V_max_ of mitochondrial complex I

A source for O_2_^•-^ formation in cells is Complex I in mitochondria (Murphy, 2009), especially following the partial inhibition of this complex (Raha et al., 2002; Murphy, 2009). We explored whether Aβ treatment reduced Complex I activity in mitochondria isolated from SH-SY5Y cells, and whether KU-32 blocked the effects of Aβ. We followed the kinetics of the rotenone-sensitive reduction of decyl-ubiquinone after addition of NADH to the mitochondria isolated from SH-SY5Y cells that were treated for 48 h with either vehicle, KU-32, Aβ_25-35_, or KU-32 plus Aβ (Figure 3B). Treatment of cells with KU-32 only led to an estimated V_max_ for Complex I activity that was significantly greater than the V_max_ of the mitochondria from vehicle control cells. Treatment of SH-SY5Y cells with Aβ significantly reduced the V_max_ of the enzyme in comparison with that of mitochondria from vehicle-treated cells. KU-32 treatment prior to exposure to Aβ reversed the effect of Aβ on the V_max_ of the enzyme and maintained the enzymatic activity at significantly higher levels than treatment of cells with vehicle only (Figure 3B). The estimated K_M_ values of Complex I in mitochondria from SH-SY5Y cells treated as described above varied within a relatively narrow range (K_M_ values, in mM, were: vehicle = 0.1; KU-32 = 0.4; Aβ = 0.07; KU-32 + Aβ = 0.2), therefore the primary effects of Aβ and KU-32 appeared to be on the V_max_ and not on the K_M_ of complex I.

To determine if the effects of KU-32 or Aβ on the V_max_ of Complex I were due to changes in the levels of protein subunits of Complex I in mitochondria from treated cells, we measured the protein levels of the NDUFB8 subunit of Complex I. This subunit, encoded in the nuclear genome, is necessary for the formation of Complex I. In mitochondrial preparations from cells exposed to vehicle, KU-32, Aβ_25-35_, and KU-32 plus Aβ, we did not detect significant differences in the levels of this subunit in the KU-32 vs. vehicle- or vs. Aβ-treated cells (data not shown). Thus, the enhancement by KU-32 of Complex I activity did not appear to be due to increased levels of Complex I proteins. Depression of the V_max_ of Complex I by Aβ without affecting the levels of Complex I proteins might result from either a direct interaction of Aβ with Complex I proteins, as suggested previously (Munguia et al., 2006), an indirect effect on a regulator of Complex I activity, or a combination of both processes.

### 3.5. KU-32 inhibition of PDHK in brain mitochondria and neuroblastoma SH-SY5Y cells

In pursuit of molecular targets of KU-32 that may be important in counteracting Aβ toxicity, we examined the effects of KU-32 on the mitochondrial enzyme pyruvate dehydrogenase kinase (PDHK). HSP90 and PDHK share a common structural feature, the C-terminal phospho-transfer domain of PDHK being a GHKL domain (Meng et al., 2014; Tso et al., 2014), similar to the GHKL domain of HSP90. There are reports of dual inhibition of Hsp90 and PDHK by non-novobiocin chemical entities (Meng et al., 2014) which prompted us to test whether KU-32 might be an inhibitor of PDHK as well as of HSP90. PDHK phosphorylates and suppresses the activity of pyruvate dehydrogenase complex (PDHC; Roche and Cate, 1977), a complex whose activity has been shown to be diminished in brains of subjects who suffered from Alzheimer’s disease (Bubber et al., 2005; Trushina et al., 2022). KU-32 inhibition of PDHK would lead to activation of PDHC of Complex I of the electron transfer chain (ETC). These observations provided the second rationale for the pursuit of studies of KU-32 on PDHK activity.

To determine the activity of PDHK in mitochondria and the effect of KU-32 on such activity, we measured the PDHC activity under conditions of no activation of PDHK, i.e., in the absence of ATP, and following PDHK activation, i.e., in the presence of ATP (Butterworth, 1989). We used isolated brain mitochondria so that we would avoid any effects that KU-32 might have on HSP90-assisted transport of pre-proteins from the cytoplasm into the mitochondria (Fan et al., 2006).

**Figure 4:**
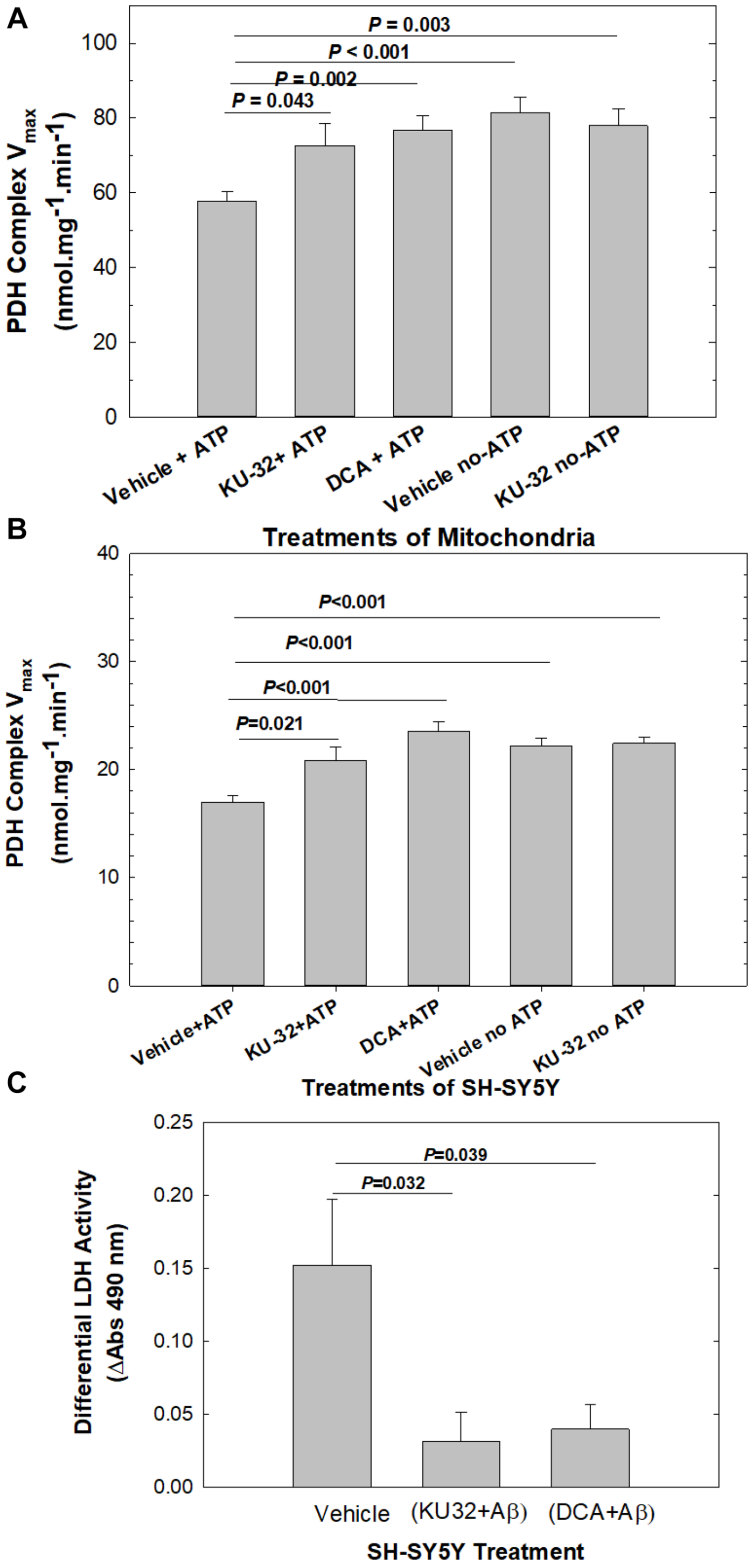
Effects of KU-32 and DCA on the regulation of PDHC activity and neuroprotection from Aβ. **(A)** Suppression by KU-32 of PDHK regulation of PDHC activity in mouse cerebellum mitochondria. Treatment of mitochondria isolated from mouse cerebellum with either vehicle (DMSO), 200 nM KU-32, or 10 mM DCA, and PDHK activated by the addition of 2 mM ATP or not activated (see Methods). Shown are the mean (± SEM) of the V_max_ of PDHC and statistically significant differences (*t*-tests). **(B)** Suppression by KU-32 of PDHK regulation of PDHC activity in homogenates derived from eight SH-SY5Y cell cultures per treatment condition. Data representation is as in **(A)**. **(C)** LDH release from SH-SY5Y cells treated for 48 h with 20 μM Aβ_25-35_ following pre-treatment by vehicle, 200 nM KU-32, or 10 mM DCA The mean (± SEM) of ΔA at 490 nm from 4 to 8 cell cultures per treatment. Values of *p* significant differences (two-tailed *t*-test).

Pre-incubation of mouse cerebellar mitochondria in an ATP-containing medium to activate PDHK (Vehicle + ATP; Figure 4A) led to a significant decrease in the V_max_ of PDHC activity in mitochondria when compared with the V_max_ of vehicle-treated mitochondria pre-incubated in the absence of ATP (Vehicle-no-ATP; Figure 4A). Mitochondria pre-treated with KU-32 and then exposed to the PDHK assay medium, in the presence (KU-32 + ATP) or absence of ATP (KU-32 no-ATP; Figure 4A), had a V_max_ for PDHC under both conditions nearly equal to that of mitochondria pre-treated with 10 mM dichloroacetate (DCA), the classic inhibitor of PDHK (DCA + ATP; Figure 4A). The V_max_ of PDHC in mitochondria pre-treated with either KU-32 or DCA and exposed to the ATP-containing PDHK assay medium did not differ significantly from that estimated for the vehicle pre-treated mitochondria exposed to the no-ATP medium, i.e., maximal activation of PDHC observed (Figure 4A). KU-32 produced very similar apparent inhibition of PDHK in mitochondria isolated from cerebral cortex homogenates as that observed for cerebellar mitochondria (data not shown). Finally, the results obtained using homogenates from neuroblastoma SH-SY5Y cells treated with KU-32, DCA, or vehicle, in the absence or presence of ATP, showed a nearly identical pattern of activation of PDHC by KU-32 and DCA as that observed with isolated brain mitochondria (Figure 4B).

Because the suppression of PDHC activity following pre-incubation of the mitochondrial preparation with Mg-ATP is an indirect measure of PDHK-induced phosphorylation of PDHC, we also analyzed the state of phosphorylation of PDHC E1α1 subunit using sandwich ELISA and a specific antibody for phospho-S232. Serine 232 is one of three serine residues of the E1α1 subunit of the enzyme phosphorylated by PDHK isoforms and is important in regulating the activity of PDHC. Treatment of mouse cerebellar mitochondria with KU-32 (200 nM) as described above, produced a significant decrease in phosphorylation of PDHC measured in the ELISA as mOD units of horseradish peroxidase substrate (tetramethylbenzidine) consumed (vehicle = 64.1 ± 3.7 vs. KU-32 = 53.5 ± 1.4, *p = 0.038*, 2-tailed test, *df* = 13). Based on these observations, we concluded that KU-32 suppressed PDHK phosphorylation of PDHC and thus enhanced the PDHC activity in mitochondria.

### 3.6. Inhibition of PDHK and neuronal protection from Aβ toxicity

To address whether inhibition of PDHK was related to neuroprotection from Aβ, we pre-treated SH-SY5Y cells with either DCA or KU-32 and measured cell viability following treatment with Aβ_25-35_. Differential LDH release from Aβ-treated cells was calculated as the net release above that from cells exposed to vehicle, from cells pre-treated with KU-32 plus Aβ as the difference from KU-32 alone, and that from cells treated with DCA plus Aβ as the difference from DCA alone (Figure 4C). Treatment with Aβ caused significantly higher levels of differential LDH release than that measured for cells pre-treated with either KU-32 plus Aβ, or DCA plus Aβ. There were no significant differences in LDH release between cells exposed to vehicle only *vs* those to KU-32 only (*p* = 0.100), or vehicle *vs* those exposed to DCA only (*p* = 0.112). Therefore, the inhibition of PDHK by either DCA or KU-32 led to neuroprotection from Aβ toxicity.

**Figure 5:**
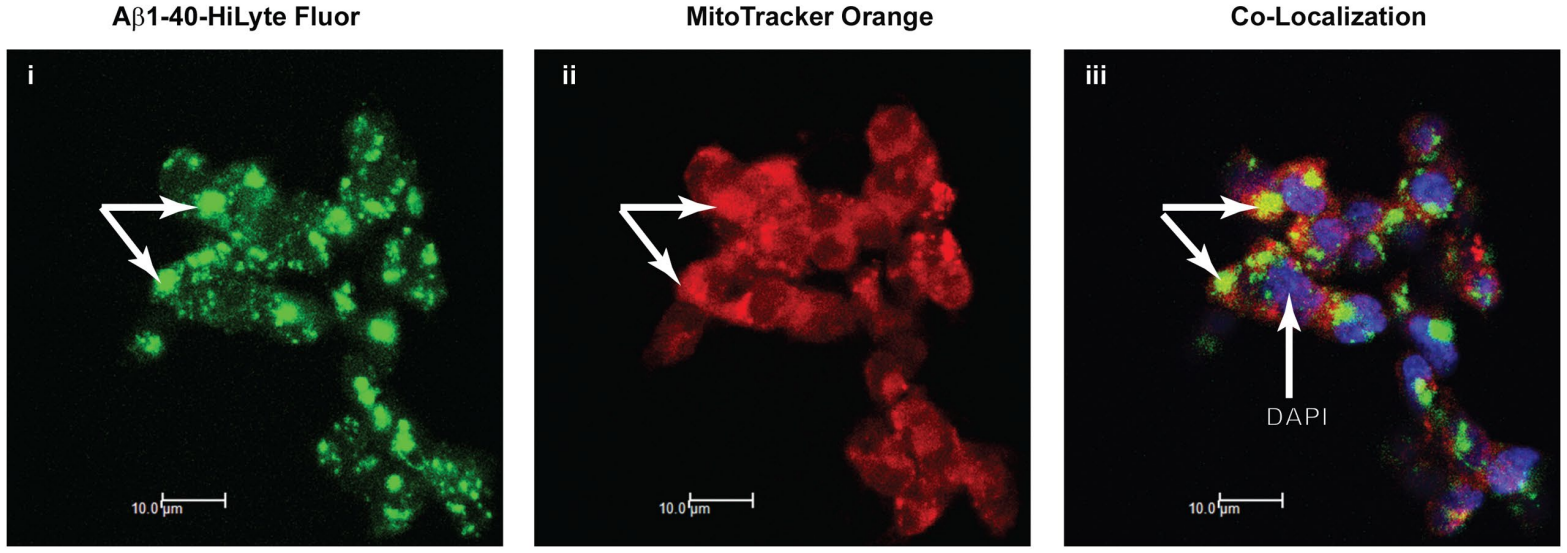
Apparent association of Aβ_1-40_ with mitochondria in SH-SY5Y cells. Immunofluorescence confocal microscopy of the distribution of Aβ_1-40_ HiLyte Fluor (I, green fluorescence), intra-mitochondrial MitoTracker (ii, red fluorescence), and the super-imposed images of these two fluorophores (yellow fluorescence) plus DAPI labeling (blue fluorescence) of nuclei (iii). Arrows point to accumulations of Aβ on MitoTracker-labeled mitochondria in cells.

The results from the studies of SH-SY5Y cells treated with Aβ were indicative of Aβ uptake or permeation into mitochondria, *in situ* accumulation in mitochondria, and interaction with the PDHK-PDHC complex in the mitochondrial matrix. Using fluorescently labeled Aβ_1-40_ as a tracer and methods developed to trace Aβ peptide accumulation in mitochondria of SH-SY5Y cells (Hansson Petersen et al., 2008), we confirmed the accumulation of fluorescently labeled Aβ_1-40_ in MitoTracker-labeled mitochondria of SH-SY5Y cells following 18 h of incubation with Aβ (Figure 5).

## 4. Discussion

The Hsp90 proteins are homo-dimers that form complexes with many co-chaperones and client proteins, creating networks that maintain proteostasis essential for cell survival (Pearl and Prodromou, 2006; Wandinger et al., 2008; Taipale et al., 2010, 2012). The discovery by Neckers (Marcu et al., 2000) that novobiocin binds weakly to a C-terminal ATP binding pocket in Hsp90 made it possible to modulate HSP90 and its complexes through a novel domain. In previous studies, we found KU-32 to be the most potent in protecting neurons against Aβ of all the novobiocin analogs synthesized at that time (Ansar et al., 2007; Lu et al., 2009). In a different model of neuronal damage, the diabetic neuropathy, KU-32 reversed the peripheral neuropathy in an HSP70-dependent manner (Urban et al., 2010; Ma et al., 2014). However, we did not see an up-regulation of HSP70 proteins in KU-32-treated primary cortical neurons. Thus, the protection of primary brain neurons against Aβ-induced toxicity by KU-32 did not correlate with changes in HSP70 levels, i.e., activation of the HSR.

The Aβ-induced neuro-toxicity has been ascribed to cellular oxidative stress and the damage to macromolecules and organelles produced by such stress (Behl, 1997; Behl and Sagara, 1997; Butterfield et al., 2007). We observed that 10 μM Aβ enhanced the levels of O_2_^•-^ in SH-SY5Ycells and that 200 nM KU-32 reduced the levels of this radical anion and diminished the effect of Aβ on O_2_^•-^ generation. Complex I of the ETC in mitochondria is a major source of O_2_^•-^ especially when Complex I is inhibited. Mitochondria from SH-SY5Y cells treated with 10 μM Aβ_25-35_ had a significantly lower V_max_ for Complex I whereas those from cells exposed to KU-32 had a significantly higher V_max_. Previous studies indicate Aβ binds selectively to a subunit of Complex I (Munguia et al., 2006) of the electron transfer chain and this protein-Aβ interaction was hypothesized to account for the partial inhibition of the enzyme by Aβ. In our studies, KU-32 enhanced the V_max_ of Complex I in mitochondria of neuroblastoma cells without affecting substantially the levels of a key organizing subunit of the complex, NDUFB8. We have not yet examined whether novobiocin analogs, such as KU-32, act by altering interactions of Aβ with specific subunits of Complex I.

An alternative mechanism for the KU-32 enhancement of Complex I V_max_ would be that KU-32 stimulates Complex I activity indirectly by enhancing the PDHC activity. As pointed out in a preceding section, HSP90 and PDHK share a common domain, the GHKL domain to which KU-32 would likely bind to PDHK and inhibit PDHK activity. PDHK phosphorylation of PDHC leads to decreases in the activity of PDHC as well as increases in O_2_^•-^ generation (Raha et al., 2002). KU-32 at 200 nM inhibited the PDHK regulation of PDHC in brain mitochondria and SH-SY5Y cells as effectively as 10 mM DCA, the well-known inhibitor of PDHK. KU-32 also partially inhibited the phosphorylation of S232 in a subunit of PDHC, the E1α1 subunit. As only the PDHK1 isoform phosphorylates S232 in the E1α1 subunit of PDHC (Korotchkina and Patel, 2001), we conclude that at a minimum one of the four isoforms of PDHK in brain is a target of KU-32.

Aβ suppression of PDHC activity in neurons, as we observed in this study, would lead to decreases in oxidative phosphorylation and to enhanced aerobic glycolysis. Aerobic glycolysis is the utilization of glucose exceeding that for oxidative phosphorylation and occurring in the presence of O_2_ levels sufficient to metabolize glucose through oxidative phosphorylation (Vaishnavi et al., 2010). Studies of human brain metabolism indicate that brain regions in which Aβ deposits accumulate in AD are regions with high rates of aerobic glycolysis (Vlassenko et al., 2010), and Aβ-induced increases in aerobic glycolysis in neurons, has been described (Santangelo et al., 2021). On the other hand, the KU-32 inhibition of PDHK would allow for the restoration of PDHC activity leading to the decarboxylation of pyruvate to form acetyl-CoA, thereby activating the Krebs cycle, the ETC, and ATP synthesis, i.e., restoration of oxidative phosphorylation and relatively normal mitochondrial function. With regard to AD, clinical severity of the disease and defects in cholinergic neurons are correlated with deficits in PDHC activity in mitochondria from AD brain (Perry et al., 1980; Sheu et al., 1985; Bubber et al., 2005), observations that support the hypothesis advanced above. Yet, others have shown that resistance of neuronal type cells to Aβ-induced toxicity increases upon activation of PDHK (Newington et al., 2012). Contrary to those observations, we found DCA-induced protection of SH-SY5Y cells from Aβ toxicity, indicating a mechanism for neuroprotection that is linked to PDHK inhibition, not activation.

Regulation of PDHC by PDHK and PDH phosphatase is very complex and depends on relative concentrations of substrates, products, co-factors, and divalent cations in mitochondria (Kohn et al., 1979). Overall, the effects of KU-32 on PDHK and Complex I activities and the neuroprotection offered by KU-32 and DCA, place the focus on mitochondrial metabolism as a determinant of Aβ-induced neurotoxicity and KU-32 neuroprotection.

Preliminary pharmacokinetic and pharmacodynamic studies of KU-32 using wild type and transgenic mice that over-express mutant tau indicate favorable penetration of KU-32 into brain and the prevention of neuronal damage in the brain of the mutant mice. Based on our observations in the present study of the neuroprotective effects of KU-32 and DCA, we propose the following scheme for the actions of Aβ in cells and for the neuroprotection offered by the novobiocin analog KU-32 (italics indicate parts of the scheme explored in the current study; bold type indicates sites or processes affected by KU-32):

Scheme→Cellular or extracellular Aβ → Cellular translocation of Aβ and entry into mitochondria **→ Activation of PDHK → Phosphorylation of PDHC → Inhibition of PDHC activity** → **Suppression of the ETC** → Decreased oxidative phosphorylation → **Increased Mitochondrial O2•-** **formation** → Mitochondrial hypo-function → Increased oxidative glycolysis.

Finally, based on the results of this study, we would suggest that inhibition of PDHK in neurons might be an appropriate target for the development of therapeutic interventions against the toxicity caused by aggregated proteins such as Aβ.

## Data availability statement

The original contributions presented in the study are included in the article/supplementary material, further inquiries can be directed to the corresponding author.

## Ethics statement

Procedures related to animals followed those of the Institutional Animal Care and Use Committee (IACUC) of the University of Kansas (IACUC # 40–09, 39–02).

## Author contributions

RP: Data curation, Investigation, Methodology, Resources, Writing – review & editing. DH: Data curation, Investigation, Methodology, Resources, Writing – review & editing. HM: Data curation, Investigation, Methodology, Writing – review & editing. HZ: Investigation, Methodology, Resources, Writing – review & editing. OM: Data curation, Methodology, Writing – review & editing. HW: Data curation, Investigation, Methodology, Writing – review & editing. BB: Conceptualization, Investigation, Resources, Writing – review & editing. CS: Data curation, Investigation, Methodology, Writing – review & editing. RS: Conceptualization, Funding acquisition, Methodology, Writing – review & editing. MM: Conceptualization, Funding acquisition, Investigation, Methodology, Writing – review & editing. EM: Conceptualization, Formal analysis, Funding acquisition, Methodology, Project administration, Writing – original draft, Writing – review & editing.

## Figures and Tables

**Table 1:** 

AD	Alzheimer’s Disease
Aβ	β-Amyloid peptide
DCA	dichloroacetic acid
DAPI	4,6-Diamidino-2-phenylindole
DMEM	Dulbecco’s modified Eagle’s medium
DMSO	dimethyl sulfoxide
DRG	Dorsal Root Ganglia
DTT	dithiothreitol
ELISA	enzyme-linked immunosorbent assay
ETC	electron transfer chain
FBS	fetal bovine serum
FCCP	carbonyl cyanide –p-trifluoromethoxyphenylhydrazone
GA	geldanamycin
HE	hydroxyethidine
Hsf1	heat shock factor 1
Hsp	Heat shock protein
HSR	heat shock response
INT	(p-iodophenyl)-3-p-nitrophenyl tetrazolium chloride
LDH	lactate dehydrogenase
O_2_•-	superoxide
2-OH-E	2-hydroxyethidium
2-OH-E+	2-hydroxyethidium
PBS	phosphate-buffered saline
PDHC	pyruvate dehydrogenase complex
PDHK	pyruvate dehydrogenase kinase
ROS	reactive oxygen species
SEM	standard error of the mean
TPP	thiamine pyrophosphate
