# Supplementary figures and images for: Protection against Aβ-induced neuronal damage by KU-32: PDHK1 inhibition as important target
Source: Front Aging Neurosci. 2023 Nov 14;15:1282855. doi: 10.3389/fnagi.2023.1282855 (PMC10682733; doi:10.3389/fnagi.2023.1282855)

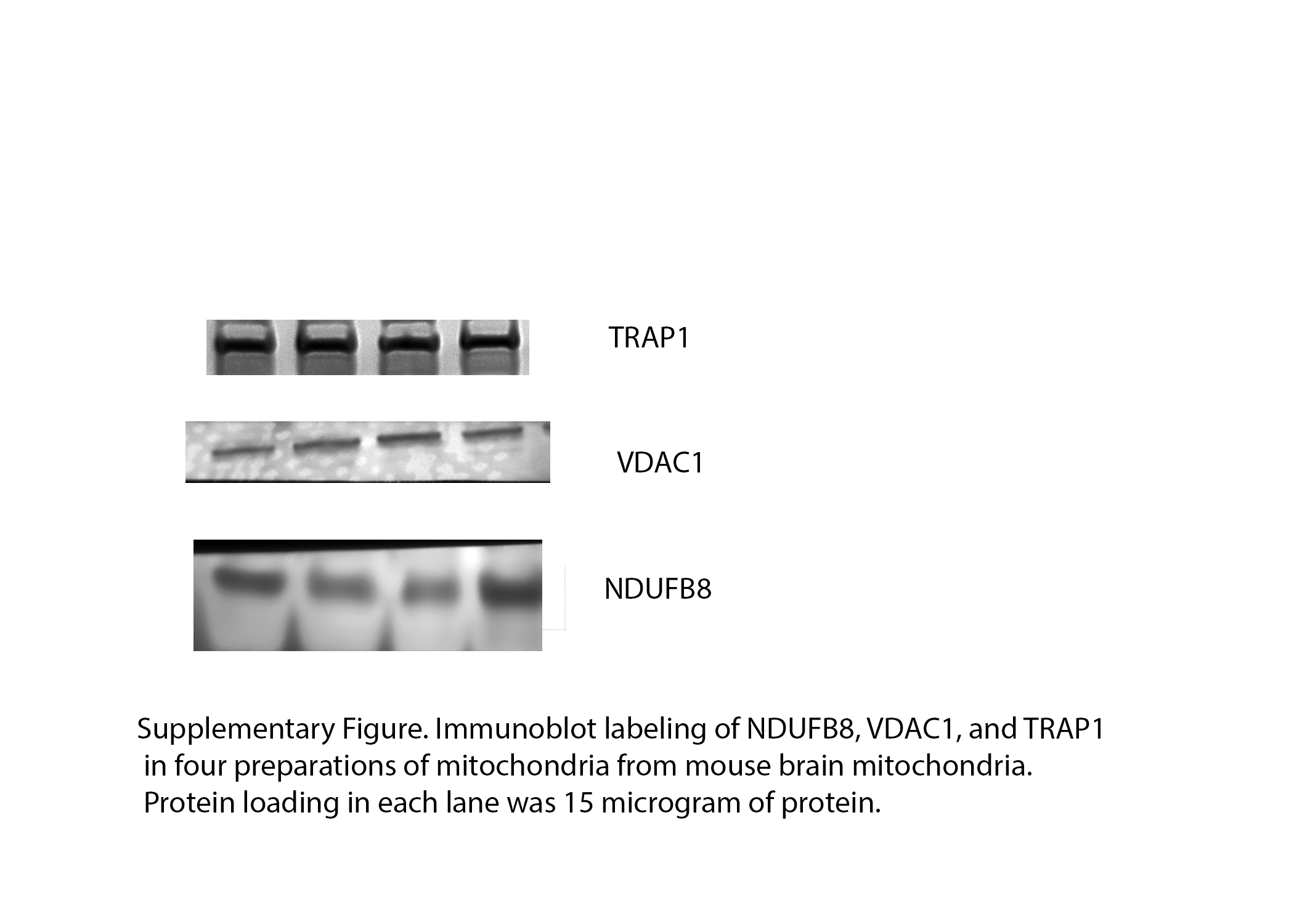

Supplement: Supplementary file 1 [file Image_1.tif]
